# Assessment of Psychopathological Comorbidities in Children and Adolescents With Autism Spectrum Disorder Using the Child Behavior Checklist

**DOI:** 10.3389/fpsyt.2019.00535

**Published:** 2019-07-26

**Authors:** Silvia Guerrera, Deny Menghini, Eleonora Napoli, Silvia Di Vara, Giovanni Valeri, Stefano Vicari

**Affiliations:** Child and Adolescence Neuropsychiatry Unit, Department of Neuroscience, Children Hospital Bambino Gesù, Rome, Italy

**Keywords:** neurodevelopmental disorders, emotional problems, behavioral problems, psychiatric comorbidity, autism spectrum disorder, Child Behavior Checklist

## Abstract

Autism spectrum disorder (ASD) is characterized by psychiatric and behavioral comorbidities. The Child Behavior Checklist (CBCL) provides valid and well-established measures of emotional, behavioral, and social problems in children and adolescents. The aim of the present study was to verify whether emotional, behavioral, and social problems were modulated by ASD symptom severity, cognitive development, gender, and age by analyzing the CBCL in a large group of children and adolescents with ASD. The results show that around 30% of participants with ASD exhibited internalizing problems and only 6% externalizing problems, with males exhibiting more internalizing problems than females. No correlation was found between CBCL scores and indices of ASD severity. However, higher CBCL Total Problems scores were found in older children and in children with lower cognitive abilities. The detection of behavioral and emotional problems allows children with ASD to undergo specific and individualized treatment that takes into account their psychopathological problems.

## Introduction

Autism spectrum disorder (ASD) is characterized by persistent deficits in social communication and social interaction across multiple contexts as well as restricted, repetitive patterns of behavior, interests, or activities (1).

Psychiatric symptoms and behavioral disorders are frequently documented in people with ASD: about two-thirds of them are indeed reported to have at least one associated mental health condition (1). Although in the past, psychiatric symptoms in children and adults with ASD have been attributed to ASD itself, an increasing number of reports suggest that additional behavior disorders in people with ASD potentially indicate the presence of psychiatric and behavioral comorbidities and demand an additional diagnosis. Attention deficit and hyperactivity disorder (ADHD), anxiety and obsessive–compulsive disorder, and emotional disorders are just some examples of psychiatric comorbidities often reported in people with ASD (2–5). It has been observed that psychiatric and behavioral comorbidities generally lead to more severe impairments because of the cumulative effects of having more than one disorder (6). Moreover, psychiatric and behavioral comorbidity can often cause more distress to caregivers than the ASD symptoms (7), interfering with help-seeking behavior and thereby affecting the long-term prognosis (8). In the field of ASD, acceptance and help-seeking behavior lead to a more positive outcome. Indeed, diagnosis and early intervention before emotional, behavioral, and social problems are firmly ingrained have significant benefits for parental mental health, such as maximizing family acceptance and adjustment to their child’s disability, and an impact on child functioning (9).

According to the *Diagnostic and Statistical Manual of Mental Disorders, Fifth Edition* (*DSM-5*) (1), additional neurodevelopmental, mental, or behavioral conditions should be specified in ASD, raising the need for a behavioral, emotional, and psychiatric evaluation. The evaluation of co-occurring disorders and follow-up examinations are then recommended and will help to determine the level of care needed.

The Child Behavior Checklist (CBCL) is a well-established and widely used parent-completed measure of emotional, behavioral, and social problems in children and adolescents aged 1.5–18 years (10, 11). It was developed to assess a range of problem behaviors (10, 11) and comprises several subscales, including Withdrawn Somatic Complaints, Anxious/Depressed, Rule-Breaking Behavior, Social Problems, Thought Problems, Attention Problems, and Aggressive Behavior. The measure provides scores for three summary scales (Internalizing, Externalizing, and Total Problems), seven or eight syndrome scales (for the age groups of 1.5–5 years and 6–18 years, respectively) representing different patterns of co-occurring emotional and behavioral problems, and five or six *DSM*-oriented scales (for the two age groups) derived through expert consensus. Each *DSM*-oriented scale reflects a broad emotional or behavioral problem that corresponds to a broad diagnostic category of the *DSM* (10). The power of the CBCL lies in a guided description of the child by the parents, whose fidelity in reporting symptoms is also widely recognized for symptoms of ASD (12).

There are a number of studies that provide data for the CBCL as a valid tool to assess co-occurring emotional and behavioral problems in children with ASD (13–17). The study by Pandolfi and colleagues (18) provides evidence to support the inclusion of the CBCL in screenings and diagnostic assessments for emotional and behavioral problems in a group of 76 youths with ASD. However, with respect to the factor validity of the CBCL, the sample size was too small to test the true hierarchical structure, and each subscale was therefore analyzed separately. The results suggest that the Total Problems scale is a valid measure for both emotional and behavioral problems, while the Internalizing Problems scale, with the Anxious/Depressed and the Withdrawn/Depressed scales, is a sensitive measure for emotional problems. Nevertheless, the authors emphasized that the CBCL had lower specificity for assessing emotional problems in their group of youths with ASD than diagnostic outcomes based on categorical classification systems (*DSM*). Recently, Magyar and Pandolfi (2) evaluated the validity of the *DSM*-oriented CBCL scales for assessing depression and anxiety in 93 children and adolescents with ASD with or without intellectual disability. The results provide strong support for using the *DSM*-oriented Affective and Anxiety Problem scales to screen emotional disorders including depression and anxiety conditions in ASD.

However, in the cited studies, the numbers of participants are small, and the patient samples constitute only a minor proportion of the general population with ASD (19–21). Moreover, the CBCL has been proposed primarily for screening of ASD in clinical settings (13, 14, 16, 17, 22, 23), rather than for evaluating psychopathological conditions and behavioral and emotional problems in comorbidity with ASD.

The aim of the present study was to assess emotional, behavioral, and social problems in relation to ASD symptom severity, cognitive development, gender, and age by analyzing the CBCL in a large group of children and adolescents with ASD.

## Material and Methods

### Participants

Seven hundred thirty-five children and adolescents were recruited for this study, between January 2008 and December 2017, at the Child and Adolescence Neuropsychiatry Unit of the Children Hospital Bambino Gesù, Rome, Italy. All children and adolescents were outpatients attending the unit for clinical assessments and rehabilitative follow-ups.

The chronological age of all participants ranged from 2.6 to 17.8 years (mean age ± sd: 10.7 ± 2.4).

Neuropsychological and psychopathological evaluations were conducted by trained developmental psychiatrists and neuropsychologists. Patients recruited earlier than 2013 received a diagnosis of ASD according to the *DSM Fourth Edition, Text Revised* (*DSM-IV-TR*) criteria (24), while those recruited later were diagnosed according to the *DSM-5* criteria (1).

### Measures

Cognitive development was assessed by the nonverbal intelligence quotient (IQ) obtained from the Leiter-R (25) or Leiter-3 (26), or by the general quotient (GQ) obtained from the Griffiths Mental Development Scales—Extended Revised for age 2–8 (GMDS-ER 2-8) (27). The Leiter-R and Leiter-3 offer a completely nonverbal measure of intelligence and evaluate the ability to reason by analogy, by matching and perceptual reasoning in general, irrespective of language and formal schooling. The brief IQ composite obtained from the Leiter-R is based on four subtests: Figure Ground, Form Completion, Sequential Order, and Repeated Patterns. Similarly, the complete IQ composite obtained from the Leiter-3 is based on four subtests: Figure Ground, Form Completion, Classification and Analogies, and Sequential Order.

The GMDS-ER 2-8 was administered when a child failed to complete the Leiter scales because of his/her reduced attentional resources. The GMDS-ER 2-8 was completed by 249 children (mean age ± sd: 4.2 ± 1.1), while the Leiter scales were completed by 153 children (mean age ± sd: 7.4 ± 3.1).

The gold-standard instruments used in this study to assess ASD symptoms were the following: the Autism Diagnostic Interview—Revised (ADI-R) (28), the Autism Diagnostic Observation Schedule—Generic (ADOS-G) (29), and the revised version ADOS-2 (30). The ADI-R is a parent-report semi-structured interview for establishing a clinical diagnosis of ASD. It follows the *DSM-IV-TR* diagnostic criteria for ASD in children with a mental age of 18 months and above. The ADI-R generates algorithm scores for each of the three subdomains of autistic symptoms: qualitative impairments in reciprocal social behavior; qualitative abnormalities in communication; and restricted range of interests and/or stereotypic behaviors. The ADOS (29, 30) is a semi-structured direct assessment of communication, social interaction, and play or imaginative use of materials for individuals with a suspected diagnosis of ASD. The ADOS consists of four (29) or five (30) modules designed for children and adults with different language levels, ranging from nonverbal to verbally fluent. The ADOS was administered and scored by licensed clinicians who have demonstrated clinical proficiency on the instrument. In the analyses, raw total scores and comparison scores (CS) were considered for the ADOS-G and ADOS-2, respectively. For the ADOS-G, the calibrated severity score (31) (renamed “CS” in the ADOS-2) was not included in the analyses, given that the present study started prior to the development of this measure.

Behavioral problems were assessed by the Achenbach System of Empirically Based Assessment (ASEBA) questionnaire. The ASEBA family of instruments is well validated to study global psychopathology in children and adolescents (32). Several ASEBA measures that allow a comprehensive approach to identify functioning in youths include the CBCL 1.5–5 years and the CBCL 6–18 years. The parents completed the CBCL; they were requested to evaluate their child’s behavior during the preceding 6 months on a 3-point Likert scale for each item (0 = not true; 1 = somewhat or sometimes true; 2 = very true or often true). All scales of the CBCL have a *t*-score mean of 50 and a standard deviation of 10, and different norms are provided for gender across age groups. The CBCL 1.5–5 comprises 100 problem items identified on several subscales, including Emotionally Reactive, Anxious/Depressed, Somatic Complaints, Withdrawn, Sleep Problems, Attention Problems, and Aggressive Behavior. Moreover, scores on Internalizing, Externalizing, and Total Problems can be obtained. The Internalizing domain is a broad measure of emotional problems. It is an aggregate of anxiety and depression symptoms that subsumes four more narrowly focused syndrome scales: Emotionally Reactive, Anxious/Depressed, Somatic Complaints, and Withdrawn. The Externalizing domain is an aggregate measure of behavioral problems and includes Attention Problems and Aggressive Behavior. The Total Problems score quantifies the overall extent of both emotional and behavioral problems, based on responses to all CBCL items including those on the one remaining syndrome scale: Sleep Problems. In the CBCL 6–18, the 113-item scale is also subdivided into several subscales, namely Withdrawn/Depressed, Somatic Complaints, Anxious/Depressed, Rule-Breaking Behavior, Social Problems, Thought Problems, Attention Problems, and Aggressive Behavior. As for the CBCL 1.5–5, scores on Internalizing, Externalizing, and Total Problems can be obtained. The Internalizing domain here subsumes three syndrome scales: Anxious/Depressed, Withdrawn/Depressed, and Somatic Complaints. The Externalizing domain includes the Rule-Breaking Behavior and Aggressive Behavior syndrome scales. The Total Problems is based on responses to all CBCL items including those on the three remaining syndrome scales: Social Problems, Thought Problems, and Attention Problems.

In the present study, Total Problems, Internalizing Problems, and Externalizing Problems scores were used as an estimate of behavioral and emotional problems. According to the normative data of the CBCL, a *t*-score ≤ 59 indicates non-clinical symptoms, a *t*-score between 60 and 64 indicates that the child is at risk for problem behaviors, and a *t*-score ≥ 65 indicates clinical symptoms (for demographical, cognitive, and psychopathological measures of participants, see **Table 1**).

**Table 1:** Demographic characteristics and cognitive and psychopathological measures of children and adolescents with autism spectrum disorder.

	N	M (SD)
Age	472	5.45 (2.94)
GQ	249	67.63 (21.27)
IQ	153	92.99 (23.29)
ADOS-G	315	13.90 (4.21)
ADOS-2	132	6.11 (1.53)
CBCL INT	472	60.50 (9.36)
CBCL EXT	472	61.34 (9.10)
CBCL TOT	472	56.47 (9.00)

GQ, general quotient; IQ, intelligence quotient; ADOS-G, Autism Diagnostic Observation Schedule—Generic total raw score; ADOS-2, Autism Diagnostic Observation Schedule-2 comparative score; CBCL INT, Child Behavior Checklist Internalizing Problems; CBCL EXT, Child Behavior Checklist Externalizing Problems; CBCL TOT, Child Behavior Checklist Total Problems.

### Data Analysis

Student’s *t*-tests were used to compare Total, Internalizing, and Externalizing Problems *t*-scores of male and female children and adolescents. To correct for multiple comparisons, the level of significance was set at *p* ≤ 0.008, using the Bonferroni correction (6 comparisons).

Pearson’s correlation coefficient was used to study the relationship between Total, Internalizing, and Externalizing Problems scores as well as measures derived from the ADOS, cognitive development, and age. To correct for multiple comparisons, the level of significance was set at *p* ≤ 0.003, using the Bonferroni correction (15 comparisons).

## Results

Out of 735 patients with ASD, the parents of 472 children completed the CBCL. Concerning Total Problems of the CBCL, the results show that 49.2% of all participants (*N* = 232) who obtained scores in the non-clinical range (*t*-score ≤ 59), 16.1% (*N* = 76) were at risk (60 ≥ *t*-score ≥ 64), and 34.7% (*N* = 164) had clinical symptoms (*t*-score ≥ 65). In sum, around 50% of all children and adolescents had a score in a clinical range or were at risk, according to their Total Problems scores. Concerning Internalizing and Externalizing Problems of the CBCL, 32.6% of all participants (*N* = 154) obtained a non-clinical score. However, as for Internalizing Problems, 13.6% (*N* = 64) of individuals were at risk, and 16.3% (*N* = 77) had clinical symptoms. Regarding Externalizing Problems, 4.2% (*N* = 20) of children were at risk, and 1.7% (*N* = 8) showed clinical scores. In sum, around 30% of participants with ASD scored in the clinical range or were at risk regarding their Internalizing Problems score, while only around 6% of participants showed Externalizing Problems (clinical or at-risk scores).

With regard to gender, 48.6% of male children with ASD (*N* = 186) obtained scores in the non-clinical range for Total Problems, 14.9% (*N* = 57) were at risk, and 36.6% (*N* = 140) showed clinical symptom scores. Of the female children, 51.7% with ASD (*N* = 46) obtained scores in the non-clinical range for Total Problems, 21.3% (*N* = 19) were at risk, and 27% (*N* = 24) showed clinical symptom scores. As for Total Problems mean scores, male and female children and adolescents did not differ (see **Table 2**).

**Table 2:** Comparisons between males and females with autism spectrum disorder on Child Behavior Checklist scales.

	Males	Females	
	M (SD)	M (SD)	*t*-value (*p*)
CBCL INT	61.88 (8.94)	59.06 (9.49)	2.65 (0.008)*
CBCL EXT	56.60 (8.92)	55.87 (9.35)	0.70 (0.48)
CBCL TOT	60.83 (9.37)	59.10 (9.24)	1.57 (0.12)

CBCL INT, Child Behavior Checklist Internalizing Problems; CBCL EXT, Child Behavior Checklist Externalizing Problems; CBCL TOT, Child Behavior Checklist Total Problems.

* The level of significance was set at p ≤ 0.008 by using Bonferroni correction (6 comparisons).

For Internalizing Problems, 35.2% of the male patients with ASD (*N* = 135) showed scores in the non-clinical range, 22.5% were at risk (*N* = 86), and 42.3% showed significant symptoms (*N* = 162). Of the female patients with ASD, 52.8% (*N* = 47) showed scores in the non-clinical range for Internalizing Problems, 16.9% were at risk (*N* = 15), and 30.3% showed clinical symptom scores (*N* = 27). Male patients showed significantly higher scores on the Internalizing Problems scale than female patients (see **Table 2**).

As for Externalizing Problems, 62.9% of male patients with ASD (*N* = 241) obtained scores in the non-clinical range, 17.2% were at risk (*N* = 66), and 19.8% (*N* = 76) showed clinical problems. Of the female patients, 60.7% (*N* = 54) obtained scores in the non-clinical range, 21.3% were at risk (*N* = 19), and 18% (*N* = 16) showed clinical problems. Male and female patients did not differ regarding their Externalizing Problems mean scores.

Analyses of the relationship between CBCL and ADOS scores did not show any significant correlation. Specifically, the Total, Internalizing, and Externalizing Problems scores of the CBCL did not correlate with the raw total score of the ADOS-G or the CS of the ADOS-2. With regard to cognitive developmental measures, the GQ was found to be inversely correlated with Total Problems scores, with participants with lower GQ showing more symptoms. The IQ was not found to correlate with Total Problems scores. No correlation was found between cognitive developmental measures (GQ or IQ) and Internalizing and Externalizing Problems scores (see **Table 3**).

**Table 3:** Correlation between Child Behavior Checklist measures and indices of autism spectrum disorder, cognitive developmental measures, and age.

	CBCL TOT		CBCL INT		CBCL EXT	
	*r*	*p*	*r*	*P*	*r*	*p*
ADOS-G	0.02	0.67	0.03	0.57	0.02	0.75
ADOS-2	−0.09	0.32	−0.07	0.45	−0.12	0.19
GQ	−0.22	0.001*	−0.17	0.007	−0.15	0.015
IQ	0.06	0.44	0.17	0.04	0.05	0.53
Age	0.2	<0.001*	0.12	0.012	0.11	0.02

CBCL TOT, Child Behavior Checklist Total Problems; CBCL INT, Child Behavior Checklist Internalizing Problems; CBCL EXT, Child Behavior Checklist Externalizing Problems; ADOS-G, Autism Diagnostic Observation Schedule—Generic total raw score; ADOS-2, Autism Diagnostic Observation Schedule-2 comparative score; GQ, general quotient; IQ, intelligence quotient.

* The level of significance was set at p ≤ 0.003 by using Bonferroni correction (15 comparisons).

Concerning the relationship between age and CBCL scores, Total Problems scores were found to correlate with age, with older children exhibiting more symptoms. No correlation was found between age and Internalizing or Externalizing Problems (see **Table 3**).

## Discussion

The present study aimed at investigating whether emotional, behavioral, and social problems were modulated by ASD symptom severity, cognitive development, gender, and age by analyzing the CBCL in a large group of 472 children and adolescents with ASD.

The CBCL is one of the most widely investigated instruments to detect emotional and behavioral problems in children and adolescents. Many studies support its reliability and validity across different clinical groups [see, for example, Ref. (33)]. Earlier investigations have corroborated its factor structure, the consistency of its major scales and subscales, and its sensitivity for identifying emotional and behavioral problems. A number of studies using the CBCL have evaluated emotional and behavioral symptoms (34, 35) as well as psychopathological comorbidities in children with neurodevelopmental disorders by examining the correlations between CBCL scores, IQ, and gender. However, the CBCL has generally been proposed for supporting an ASD diagnosis in a clinical setting (18, 36), while very little is known about the development of internalizing and externalizing problems in isolation, and nothing is known about their joint development (37).

In the present study, CBCL scores were correlated with indices of ASD severity, cognitive developmental measures, and age. Differences between male and female patients regarding Total, Internalizing, and Externalizing Problems scores of the CBCL were also evaluated.

Our results show that out of the 472 examined children, around 30% exhibited Internalizing Problems, with CBCL scores in the clinical or at-risk range, and with significantly higher scores for male than female children. However, concerning Externalizing Problems, only 6% of all participants had clinical or at-risk scores.

Similarly, an earlier study by Hartley and colleagues (22) reported that 29.6% of children with ASD had significant Internalizing Problems. However, the authors reported a higher percentage for Externalizing Problems (27.2%) than what we found in the current study. This discrepancy could be due to differences in participant characteristics, with younger children (mean age 3.5 years) involved in the study by Hartley and colleagues (20) and older children in our study (mean age 10.7 years old). Indeed, the Externalizing Problems scale is derived from Attention Problems and Aggressive Behavior items that may be displayed more prominently at younger ages.

A previous report (38) studied phenotypic expressions of behavioral problems in 215 preschool children (3 years old) by using CBCL in different clinical populations and typically developing children. The authors evidenced that children with ASD or cerebral palsy had more total symptoms and more internalizing problems than children with Down syndrome or typically developing children. Moreover, children with ASD showed more externalizing symptoms than typically developing children. A reduction of externalizing symptoms during early childhood in ASD was also documented. This result could explain why a small percentage of externalizing symptoms was found in our participants with mean age of 10.7 years old.

Psychiatric and behavioral comorbidities were also examined in studies that assessed the *DSM*-oriented subscale of CBCL (39) or single clinical subscales (40, 41). In particular, Georgiades and colleagues (40) explored in 335 preschool children with ASD the following CBCL subscales: Emotionally Reactive, Anxious/Depressed, Somatic Complaints, Withdrawn, Attention Problems, and Aggressive Behavior. Results showed that 39.2% of children with ASD were in the “clinical range” on the Withdrawn subscale, whereas 11.7% and 7.2% scored above the clinical cutoff on the Attention Problems and Emotionally Reactive subscales, respectively. In this preschool-age population, the study (40) documented higher prevalence of internalizing symptoms than externalizing symptoms.

Llanes and colleagues (41) used the CBCL (Parent and Teacher Report Forms) to evaluate the prevalence of ADHD symptoms and anxiety in 180 preschools (age range 4–5 years) and school-aged children (age range 6–7 years) with ASD. In the study, parents reported anxiety symptoms in 31% of preschool children and in 50% of school-aged children and ADHD symptoms in 22% of preschool children and 45% of school-aged children. Even if authors did not report specific scores for Internalizing and Externalizing Problems scales, the study (41) showed more anxiety symptoms than ADHD symptoms at each age investigated, documenting a high percentage of internalizing problems. Moreover, in our study, children with ASD were older (mean age 10.7 years) than those in the study by Llanes and colleagues (41), and it could be that internalizing symptoms are more present in older than in younger children.

There are studies that did not use CBCL to investigate behavioral and emotional problems in individuals with ASD but adopted different diagnostic approaches and assessment batteries. Thus, it is difficult to compare our results with these studies, when researchers use different tools to assess comorbid psychopathology symptoms and diagnose psychiatric disorders.

For example, concerning an internalizing disorder such as anxiety, the prevalence rates of anxiety in children with ASD ranged from 11% to 84% in a literature review (42, 43).

The review by Skokauskas and Callager (44) highlighted that anxiety disorders represent the most common psychiatric comorbidity in individuals with ASD. For example, Caamaño and colleagues (45) found that 76% of children and adolescents with ASD presented anxiety symptoms and 56% attention problems; Amr and colleagues (46) found that 58.3% of children with ASD showed anxiety disorders and 13.3% a major depressive disorder (while only 31.6% and 23.3% presented ADHD and conduct disorders, respectively).

Although internalizing symptoms were frequently found associated with ASD, a wide range of externalizing symptoms were also documented in ASD. Sizing and colleagues (47) carried out a study on ADHD-like symptoms using 3 rating scales. Results revealed a high phenotypical overlap between ASD and ADHD (53%) that was interpreted by authors as the effect of difficulties in communicating needs and in attracting adult attention in children with ASD. Another study (5) used a parent interview (the Child and Adolescent Psychiatric Assessment) to assess emotional and behavioral problems in 112 children with ASD (age range 10–14 years). Results evidenced that the most common diagnoses were social anxiety disorder (29.2%), ADHD (28.2%), and oppositional defiant disorder (28.1%). Even if authors (5) found a high percentage of internalizing disorders (such as social anxiety disorder), it is difficult to compare their results with our results obtained by CBCL, which did not consider *DSM*-oriented scales and individual subscales.

Despite the different method adopted (the Problem Behavior scale of the Scales of Independent Behavior—Revised) (48), Shattuck and colleagues (49) examined internalizing and externalizing problems across 4.5 years in 241 individuals with ASD with a wide age range (from 10 years to 52 years). Similarly to our results, the authors found a higher prevalence of internalizing behaviors (hurtful to self, unusual or repetitive habits, withdrawal or inattentive behavior) than externalizing behaviors (hurtful to others, destructive to property, disruptive behavior) in individuals with ASD.

In our study, no correlation was found between CBCL scores and indices of ASD severity (ADOS-G raw total score and ADOS-2 CS), similarly to previous results that showed no relation (20).

Conversely, a correlation was observed between age and CBCL scores, with older children exhibiting more symptoms for Total Problems than younger ones. The higher percentage of behavioral problems in older children with ASD could be interpreted in the light of increasing social and environmental demands and resulting difficulties of older children in adjusting their behavior to changing environmental demands. Since the CBCL is completed by the parents, another possible interpretation is that parents are more likely to have concerns regarding emotional and behavioral problems when their children are older. It is difficult, however, to compare our results with previous studies on the relation between age and CBCL scores, due to differences in analysis methods between studies. For example, the study by Hartley and colleagues (20) did not find a significant correlation between age and CBCL Externalizing Problems scores, but it documented a positive correlation between age and Internalizing Problems scores. Since the authors did not consider Total Problems scores in their analyses, it is difficult to compare our results with their findings (20).

A negative correlation was also found between CBCL Total Problems scores and cognitive developmental measures as evaluated by the GMDS-ER 2-8, since children with ASD with lower GQ had higher Total Problems scores. However, no correlation was found between IQ measured by the Leiter scales and CBCL Total Problems scores. This result could be explained by differences in the mean age of children evaluated using the GMDS-ER 2-8 and the Leiter scales. Indeed, the former group evaluated using the GMDS-ER 2-8 was younger (mean age 4.2 years) than the second group (mean age 7.4 years) evaluated using the Leiter scales. An early study conducted by Bölte and colleagues (19) in 77 children with ASD (54 male and 23 female children, with a mean age of 11.3 years) found a significant influence of age and IQ on CBCL scores, with no gender differences in CBCL scores. It is, however, difficult to compare our results with the findings by Bölte and colleagues (19), since their CBCL scores were not normalized and their analyses were conducted on raw scores. More recently, Hartley and colleagues (20) studied correlations between CBCL Externalizing Problems, Internalizing Problems, and syndrome scale scores and characteristics such as cognitive functioning, gender, age, ethnicity, expressive language, and severity of autistic and adaptive behaviors in 169 children with ASD (mean age 3.5 years). Similar to our results in a younger group of participants, they found that CBCL scores correlated with cognitive functioning scores, with a significant negative correlation between Internalizing and Externalizing Problems scores and cognitive functioning measures. Mensi and colleagues (21) also evaluated correlations between emotional and behavioral problems (derived from the CBCL 1.5–5 years) and the GQ (derived from the GMDS-ER 2-8), age, and ADOS and ADI-R scores in a group of 90 children with ASD with a mean age of 3.9 years who were diagnosed according to the *DSM-IV* criteria. Mothers and fathers completed the CBCL separately, and the authors showed a significant low-grade positive correlation between the children’s age and their CBCL scores on the Pervasive Developmental Disorders scale of the questionnaires completed by the mothers. No correlation with age was found in the questionnaires completed by the fathers. In the CBCL completed by both mothers and fathers, some positive correlations were identified between the Internalizing, Withdrawal, and Pervasive Developmental Disorders scores and the subscales relating to social interactions and behavior of the ADI-R. Beside these measures to confirm a diagnosis of ASD, the findings by Mensi and colleagues (21) on a relationship between CBCL scores and GMDS-ER 2-8 measures are in line with our results. An inverse correlation between the GMDS-ER 2-8 Language subscale and Total and Externalizing Problems scores for the fathers and Total Problems scores for the mothers was found. Furthermore, the study by Mensi and colleagues (21) emphasizes the importance of considering the different perspectives of the mother and the father to obtain a complete representation of the emotional and behavioral problems of the child.

The present study underlines the need to use instruments such as the CBCL in children with ASD, not just for screening and forming a diagnosis, but also for detecting emotional and behavioral problems. Indeed, our results point out that psychopathological comorbidities (especially internalizing symptoms) are present in a significant number of children with ASD (in 30% of the population investigated) and that they are related to gender (more present in males), age (more present in older children), and cognitive development (more present in children with lower GQ).

The detection of related conditions in ASD is a major concern for experts in the field (50). The inherent heterogeneity of ASD, with a variety of different symptom clusters, complicates the goal of identifying specific treatments. Several factors make it difficult to assess emotional and behavioral problems in many individuals with ASD. These include apparent symptom overlap between characteristics of ASD and symptoms of comorbidities (51). In recent years, there is a growing recognition of the need for individualization of treatment and approaches in ASD (52), and the detection of behavioral and emotional problems allows children with ASD to undergo more specific and individualized treatments that take into account their psychopathological problems.

We believe this study with a large sample size helps move the field toward more precise and valid measurements of psychopathologies in young children and adolescents with ASD, and our findings have implications for the quality of life and long-term outcomes of those individuals. The present study has several limitations. First, since this is a retrospective study, a cause-and-effect relationship cannot be determined, but it is useful for providing preliminary data and in guiding the development of future prospective studies. Clinical assessments of ASD were conducted over a period of 9 years, using different instruments to asses ASD such as the ADOS-G and the revised version ADOS-2. Therefore, analyses required us to include two different scores: raw total scores for the ADOS-G and comparison scores for the ADOS-2. Moreover, we analyzed data that were collected prior to the development of the calibrated severity score (included only in the ADOS-2), and it was thus not possible to consider this single valid measure to more accurately capture the core symptom severity of ASD.

Second, our analyses examined only three main areas of the CBCL questionnaire (Total Problems, Internalizing Problems, Externalizing Problems). By examining only these three scales, we did not analyze more discrete aspects of emotional and behavioral problems, such as individual subscales or *DSM*-oriented scales. Further studies are needed to assess the emotional and behavioral profile of children with ASD by using more CBCL scales and more objective assessment of psychiatric comorbidity.

Moreover, the CBCL questionnaire was completed by only one parent, while studies that analyze reports from both parents may be more informative.

Future studies should also examine emotional and behavioral problems of children with ASD against a typically developing control group or other group with neurodevelopmental disorders to control for the nonspecific psychopathological comorbidity in ASD.

Finally, it’s difficult to distinguish how much of clinical scores on CBCL are affected by core ASD symptoms or by comorbid psychiatric, emotional, and behavioral problems. However, Hess and colleagues (53) documented that internalizing symptoms (worry/depressed, undereating, overeating, avoidant behavior, and repetitive behavior) should be taken into account in ASD since they were significantly different between individuals with and without ASD, whereas externalizing symptoms (conduct behavior and tantrum behavior) were not significantly different among those with and without ASD. Noordhof and colleagues (54) found that ASD-related problems assessed by CBCL constituted a specific domain of psychopathology that could be distinguished from the internalizing and externalizing.

In conclusion, our study assessed psychopathological comorbidities in a large group of children with ASD. Since psychiatric disorders can worsen symptoms of ASD, interfere with education, and reduce the benefits of treatment, more studies on the detection of these problems are needed and will allow children with ASD to receive appropriate therapies that take into account their emotional and behavioral problems.

## Ethics Statement

All procedures performed in studies involving human participants were in accordance with the ethical standards of the institutional and/or national research committee and with the 1964 Declaration of Helsinki and its later amendments or comparable ethical standards. For this type of study, formal consent is not required. Written informed consent was obtained from parents of each participant included in the study.

## Author Contributions

SG participated in the design and coordination of the study and interpretation of the data, performed the measurement, and drafted and revised the manuscript. DM conceived of the study, participated in its design and interpretation of the data, performed the statistical analysis, and helped to draft and revise the manuscript. EN participated in the design of the study, performed the measurement, and helped to draft and revise the manuscript. SD participated in the design of the study, performed the measurement, and helped to draft and revise the manuscript. GV participated in the design of the study and helped to draft the manuscript. SV participated in the design of the study, coordination, and interpretation of the data, and helped to draft the manuscript. All authors read and approved the final manuscript.

## Conflict of Interest Statement

The authors declare that the research was conducted in the absence of any commercial or financial relationships that could be construed as a potential conflict of interest.

## References

[1] American Psychiatric Association Diagnostic and statistical manual of mental disorders (DSM-5 ^®^). Washington: American Psychiatric Publishing (2013). 10.1176/appi.books.9780890425596

[2] Magyar CI Pandolfi V Utility of the CBCL *DSM*-oriented scales in assessing emotional disorders in youth with autism. Res Autism Spectr Disord (2017) 37:11–20. 10.1016/j.rasd.2017.01.009 28970862 PMC5621768

[3] Romero M Aguilar JM Del-Rey-Mejías Á Mayoral F Rapado M Peciña M Psychiatric comorbidities in autism spectrum disorder: a comparative study between *DSM-IV-TR* and *DSM-5* diagnosis. Int J Clin Health Psychol (2016) 16:266–75. 10.1016/j.ijchp.2016.03.001 PMC6225088 30487870

[4] van Steensel FJ Bögels SM Perrin S Anxiety disorders in children and adolescents with autistic spectrum disorders: a meta-analysis. Clin Child Fam Psychol Rev (2011) 14:302. 10.1007/s10567-011-0097-0 21735077 PMC3162631

[5] Simonoff E Pickles A Charman T Chandler S Loucas T Baird G Psychiatric disorders in children with autism spectrum disorders: prevalence, comorbidity, and associated factors in a population-derived sample. J Am Acad Child Adolesc Psychiatry (2008) 47:921–9. 10.1097/CHI.0b013e318179964f 18645422

[6] Gadow KD Guttmann-Steinmetz S Rieffe C DeVincent CJ Depression symptoms in boys with autism spectrum disorder and comparison samples. J Autism Dev Disord (2012) 42:1353–63. 10.1007/s10803-011-1367-x 21960455

[7] Lecavalier L Leone S Wiltz J The impact of behaviour problems on caregiver stress in young people with autism spectrum disorders. J Intellect Disabil Res (2003) 50:172–83. 10.1111/j.1365-2788.2005.00732.x 16430729

[8] Horner RH Diemer SM Brazeau KC Educational support for students with severe problem behaviors in Oregon: a descriptive analysis from the 1987–88 school year. J Assoc Pers Sev Handicaps (1992) 17:154–69. 10.1177/154079699201700304

[9] Daley TC From symptom recognition to diagnosis: children with autism in urban India. Soc Sci Med (2004) 58:1323–35. 10.1016/S0277-9536(03)00330-7 14759679

[10] Achenbach TM Rescorla L Manual for the ASEBA preschool forms and profiles. Burlington, VT: University of Vermont, Research center for children, youth, & families (2000).

[11] Achenbach TM Rescorla L Manual for the ASEBA school-age forms & profiles. Burlington, VT: University of Vermont, Research center for children, youth, & families (2001).

[12] So P Greaves-Lord K van der Ende J Verhulst FC Rescorla L de Nijs PF Using the Child Behavior Checklist and the teacher’s report form for identification of children with autism spectrum disorders. Autism (2013) 17:595–607. 10.1177/1362361312448855 22914776

[13] Biederman J Petty CR Fried R Wozniak J Micco JA Henin A Child Behavior Checklist clinical scales discriminate referred youth with autism spectrum disorder: a preliminary study. J Dev Behav Pediatr (2010) 31:485–90. 10.1097/DBP.0b013e3181e56ddd 20585266

[14] Ooi YP Rescorla L Ang RP Woo B Fung DSS Identiﬁcation of autism spectrum disorders using the Child Behavior Checklist in Singapore. J Autism Dev Disord (2011) 41:1147–56. 10.1007/s10803-010-1015-x 20405192

[15] Achenbach TM Rescorla L Achenbach System of Empirically Based Assessment. In: Volkmar FR, editor. Encyclopedia of autism spectrum disorders. NY: Springer (2013). p. 31–9. 10.1007/978-1-4419-1698-3_219

[16] Muratori F Narzisi A Tancredi R Cosenza A Calugi S Saviozzi I The CBCL1.5–5 and the identiﬁcation of preschoolers with autism in Italy. Epidemiol Psychiatr Sci (2011) 20:329–38. 10.1017/S204579601100045X 22201210

[17] Narzisi A Calderoni S Maestro S Calugi S Mottes E Muratori F Child behavior check list 1½–5 as a tool to identify toddlers with autism spectrum disorders: a case–control study. Res Dev Disabil (2013) 34:1179–89. 10.1016/j.ridd.2012.12.020 23376628

[18] Pandolfi V Magyar CI Norris M Validity study of the CBCL 6–18 for the assessment of emotional problems in youth with ASD. J Ment Health Res Intellect Disabil (2014) 7:306–22. 10.1080/19315864.2014.930547 PMC4239123 25419257

[19] Bölte S Dickhut H Poustka F Patterns of parent-reported problems indicative in autism. Psychopathology (1999) 2:93–7. 10.1159/000029072 10026453

[20] Hartley SL Sikora DM McCoy R Prevalence and risk factors of maladaptive behaviour in young children with autistic disorder. J Intellect Disabil Res (2008) 52:819–29. 10.1111/j.1365-2788.2008.01065.x PMC2838711 18444989

[21] Mensi MM Carigi T Chiappedi M Balottin U Correlazioni tra CBCL e variabili cliniche e socio-demografiche in bambini con Disturbi Pervasivi dello Sviluppo. Boll Soc Med Chir di Pavia (2013) 126:571–7. 10.6092/2039-1404.126.1585

[22] Duarte CS Bordin IA de Oliveira A Bird H The CBCL and the identification of children with autism and related conditions in Brazil: pilot findings. J Autism Dev Disord (2003) 33:703–7. 10.1023/B:JADD.0000006005.31818.1c 14714937

[23] Havdahl KA von Tetzchner S Huerta M Lord C Bishop SL Utility of the Child Behavior Checklist as a screener for autism spectrum disorder. Autism Res (2016) 9:33–42. 10.1002/aur.1515 26140652 PMC4939629

[24] American Psychiatric Association Diagnostic and statistical manual of mental disorders 4^th^ ed., text revised (DSM-IV-TR). Washington, DC: American Psychiatric Association (2000).

[25] Roid GH Miller LJ Leiter International Performance Scale—Revised. Woodale, IL: Stoelting C (1997). 10.1037/t05120-000

[26] Roid GH Miller LJ Pomplun M Koch C Leiter International Performance Scale, Third Edition (Leiter-3). Los Angeles: Western Psychological Services (2013). Italian edition Cornoldi C Giofrè D Belacchi C, Editors. Firenze: Giunti Organizzazioni Speciali.

[27] Luiz D Barnard A Knoesen N Kotras N Horrocks S McAlinden P GMDS-ER 2-8—Griffiths Mental Development Scales—Extended Revised: 2 to 8 years (2006). Italian edition Cianchetti C Fancello GS, Editors. Firenze: Giunti Organizzazioni Speciali.

[28] Lord C Rutter M Le Couteur A Autism Diagnostic Interview—Revised: a revised version of a diagnostic interview for caregivers of individuals with possible pervasive developmental disorders. J Autism Dev Disord (1994) 24:659–85. 10.1007/BF02172145 7814313

[29] Lord C Risi S Lambrecht L Cook EH Leventhal BL DiLavore PC The Autism Diagnostic Observation Schedule—Generic: a standard measure of social and communication deficits associated with the spectrum of autism. J Autism Dev Disord (2000) 30:205–23. 10.1023/A:1005592401947 11055457

[30] Lord C Rutter M DiLavore PC Risi S Gotham K Bishop S Autism Diagnostic Observation Schedule—Second Edition (ADOS-2). Los Angeles: Western Psychological Service Italian edition. Colombi C Tancredi R Persico A Faggioli A, Editors. Firenze: Hogrefe Editore (2012).

[31] Gotham K Pickles A Lord C Standardizing ADOS scores for a measure of severity in autism spectrum disorders. J Autism Dev Disord (2009) 39:693–705. 10.1007/s10803-008-0674-3 19082876 PMC2922918

[32] Rescorla LA Assessment of young children using the Achenbach System of Empirically Based Assessment (ASEBA). Dev Disabil Res Rev (2005) 11:226–37. 10.1002/mrdd.20071 16161094

[33] Bérubé RL Achenbach TM Bibliography of published studies using the Achenbach System of Empirically Based Assessment (ASEBA). Burlington, VT: University of Vermont, Research Center for Children, Youth, and Families (2010).

[34] Osório AA Rossi NF Gonçalves ÓF Sampaio A Giacheti CM Psychopathology and behavior problems in children and adolescents with Williams syndrome: distinctive relationships with cognition. Child Neuropsychol (2017) 23:631–41. 10.1080/09297049.2016.1183607 27224940

[35] Rietman AB Oostenbrink R van Noort K Franken MCJ Catsman-Berrevoets CE Aarsen FK Development of emotional and behavioral problems in neurofibromatosis type 1 during young childhood. Am J Med Genet A (2017) 173:2373–80. 10.1002/ajmg.a.38323 28627810

[36] Pandolfi V Magyar CI Dill CA An initial psychometric evaluation of the CBCL 6–18 in a sample of youth with autism spectrum disorders. Res Autism Spectr Disord (2012) 6:96–108. 10.1016/j.rasd.2011.03.009 22059091 PMC3207215

[37] Vaillancourt T Haltigan JD Smith I Zwaigenbaum L Szatmari P Fombonne E Joint trajectories of internalizing and externalizing problems in preschool children with autism spectrum disorder. Dev Psychopathol (2017) 29:203–14. 10.1017/S0954579416000043 26847324

[38] Eisenhower AS Baker BL Blacher J Preschool children with intellectual disability: syndrome specificity, behaviour problems, and maternal well-being. J Intellect Disabil Res (2005) 49:657–71. 10.1111/j.1365-2788.2005.00699.x PMC3072759 16108983

[39] Kanne SM Abbacchi AM Constantino JN Multi-informant ratings of psychiatric symptom severity in children with autism spectrum disorders: the importance of environmental context. J Autism Dev Disord (2009) 39:856–64. 10.1007/s10803-009-0694-7 PMC2878186 19191016

[40] Georgiades S Szatmari P Duku E Zwaigenbaum L Bryson S Roberts W Phenotypic overlap between core diagnostic features and emotional/behavioral problems in preschool children with autism spectrum disorder. J Autism Dev Disord (2011) 41:321–9. 10.1007/s10803-010-1158-9 21161575

[41] Llanes E Blacher J Stavropoulos K Eisenhower A Parent and teacher reports of comorbid anxiety and ADHD symptoms in children with ASD. J Autism Dev Disord (2018) 1–12. 10.1007/s10803-018-3701-z 30062398

[42] Grondhuis SN Aman MG Assessment of anxiety in children and adolescents with autism spectrum disorders. Res Autism Spectr Disord (2012) 6:1345–65. 10.1016/j.rasd.2012.04.006

[43] White SW Oswald D Ollendick T Scahill L Anxiety in children and adolescents with autism spectrum disorders. Clin Psychol Rev (2009) 29:216–29. 10.1016/j.cpr.2009.01.003 PMC2692135 19223098

[44] Skokauskas N Gallagher L Mental health aspects of autistic spectrum disorders in children. J Intellect Disabil Res (2012) 56:248–57. 10.1111/j.1365-2788.2011.01423.x 21554467

[45] Caamaño M Boada L Merchán-Naranjo J Moreno C Llorente C Moreno D Psychopathology in children and adolescents with ASD without mental retardation. J Autism Dev Disord (2013) 43:2442–9. 10.1007/s10803-013-1792-0 23468070

[46] Amr M Raddad D El-Mehesh F Bakr A Sallam K Amin T Comorbid psychiatric disorders in Arab children with autism spectrum disorders. Res Autism Spectr Disord (2012) 6:240–8. 10.1016/j.rasd.2011.05.005

[47] Sinzig J Walter D Doepfner M Attention deficit/hyperactivity disorder in children and adolescents with autism spectrum disorder: symptom or syndrome? J Atten Disord (2009) 13:117–26. 10.1177/1087054708326261 19380514

[48] Bruininks RH Woodcock RW Weatherman RF Hill BK Scales of Independent Behavior—Revised. USA: Riverside Publishing (1996).

[49] Shattuck PT Seltzer MM Greenberg JS Orsmond GI Bolt D Kring S Change in autism symptoms and maladaptive behaviors in adolescents and adults with an autism spectrum disorder. J Autism Dev Disord (2007) 37:1735–47. 10.1007/s10803-006-0307-7 PMC3265360 17146700

[50] Committee on Children with Disabilities. The pediatrician’s role in the diagnosis and management of autistic spectrum disorder in children. Pediatrics (2001) 107:1221–6. 10.1542/peds.107.5.1221 11331713

[51] Stewart ME Barnard L Pearson J Hasan R O’Brien G Presentation of depression in autism and Asperger syndrome: a review. Autism (2006) 10:103–16. 10.1177/1362361306062013 16522713

[52] Sherer MR Schreibman L Individual behavioral profiles and predictors of treatment effectiveness for children with autism. J Consult Clin Psychol (2005) 73:525. 10.1037/0022-006X.73.3.525 15982150

[53] Hess JA Matson JL Dixon DR Psychiatric symptom endorsements in children and adolescents diagnosed with autism spectrum disorders: a comparison to typically developing children and adolescents. J Dev Phys Disabil (2010) 22:485–96. 10.1007/s10882-009-9185-1

[54] Noordhof A Krueger RF Ormel J Oldehinkel AJ Hartman CA Integrating autism-related symptoms into the dimensional internalizing and externalizing model of psychopathology. The TRAILS Study. J Abnorm Child Psychol (2014) 43:577–87. 10.1007/s10802-014-9923-4 25099360

